# Language ideologies in transnational families with Israeli background in Finland

**DOI:** 10.3389/fsoc.2024.1453226

**Published:** 2024-10-09

**Authors:** Gali Bloch

**Affiliations:** Department of Languages, University of Helsinki, Helsinki, Finland

**Keywords:** language ideologies, transnational family, Nordic countries, Modern Hebrew, Generation 1.5

## Abstract

Transnational families manage complex language dynamics, with multifaceted linguistic practices representing a key aspect in shaping communication among family members, where balancing heritage and host country languages impacts both cultural preservation and integration into a new society. The situation complicates when multilingual and multicultural families relocate to a country with a new majority language, while maintaining ties to their original society. This study analyzes language ideologies of seven transnational participants who were repatriated to Israel from post-Soviet states (PSS) during childhood and decades later relocated to Finland with their children. The study based on the data gathered through semi-structured interviews addresses two key questions: What are the language ideologies held by Israeli Generation 1.5 parents in transnational multilingual families residing in Finland? What are the parental language beliefs concerning their and their children's social integration in Finland? Thematic data analysis reveals parents' efforts to balance multiple languages, driven by their overwhelmingly positive views on their children's multilingualism. The language choices impact language acquisition and maintenance, while also preserving old social connections and building new ones for the entire family. Findings highlight key factors shaping parental ideologies on language transmission, multilingualism, code-switching, and integration, mostly influenced by personal convenience and practicality. This study presents a novel perspective on the language ideologies of multilingual parents. By examining individual parental beliefs and attitudes toward each language involved, it identifies recurring collective ideologies regarding multilingualism overall and each specific language, thus enriching discussions on linguistic diversity and multicultural integration in transnational contexts. Additionally, informing policymakers about the linguistic challenges encountered by transnational multilingual families can facilitate the promotion of inclusive educational practices and foster cultural awareness initiatives, thus contributing to the creation of a more equitable and supportive environment for such families managinglinguistic diversity.

## 1 Introduction

Language plays a pivotal role in parenting, especially in an immigrant context, where parents face a number of linguistic challenges in communicating with their children (Lanza, 2021). These challenges involve decisions about which language(s) to use for effective communication and social integration, the degree to which the heritage language (HL) should be supported, and how (or whether) to balance the environmental language(s) with the HL(s). The situation becomes even more complex when the parents themselves are balanced bi- or multilinguals, and need to make choices concerning which of their native languages to transmit to their children in a new linguistic environment. The patterns and strategies for language choices and usage in trilingual and multilingual families are not yet well-understood, with findings varying significantly across different studies. Most research are case studies, making it difficult to discern general patterns (Quirk et al., 2024), and more ethnographically informed studies “examining the intricacies of these two poles” (family language ideologies and actual translingual practices at home) “are needed to continually examine successful ways of managing multilingualism in the home environment” (Soler and Zabrodskaja, 2017). In general, the absence of larger datasets of trilingual families makes it difficult to predict clear patterns and make generalizations.

In the European Union, the main or official language(s) of the environment is usually the language used in schools (Dockrell et al., 2022). Many children also speak minority or HLs often excluded from educational and public contexts, making these languages available almost exclusively at home. The acquisition of a minority language at home primarily relies on the decisions, whether deliberate or spontaneous, made by the children's parents and permanent caregivers. Such linguistic choices are heavily influenced by parental language ideologies, as well as by societal factors (De Houwer, 2021). This study advances the understanding of parental language choices by focusing on a previously underexplored language combination. It provides insights into the distinctive features of a trilingual language environment, examining the language ideologies of transnational families with bilingual Russian-Hebrew parents raising their children in a third language context. Seven parents were interviewed to investigate their family language policies, especially parental ideologies, toward multilingualism in their children. The interviews also aimed to understand parental beliefs and attitudes toward the factors affecting the successful transmission or attrition of each language involved. The research questions were: What are the language ideologies held by Israeli Generation 1.5 parents in transnational multilingual families residing in Finland? What are the parental language beliefs concerning their and their children's social integration in Finland?

This article is organized as follows. The theoretical background section explores language ideologies concerning HL transmission in transnational multilingual families in general, and the language ideologies of Israeli Generation 1.5 Russian-Hebrew speakers in particular. The Methodology and Data section provides a detailed description of the demographic and linguistic dimensions of the Israeli diaspora, as well as the data analysis framework. The following section presents the findings from interviews conducted for the study. The final section discusses the key outcomes of the investigation summarized in the conclusion.

## 2 Background

### 2.1 Theoretical background: language ideologies

The concept of “linguistic ideologies” was introduced by Silverstein (1979), who defined them as “sets of beliefs about language articulated by users as a rationalization or justification of perceived language structure and use” (Silverstein, 1979, p. 193). Language ideologies, as described by Schiffman (1998), encompass cultural assumptions and underlying policies regarding language, correctness, and preferred modes of communication. Language correctness in this paper follows Spolsky's definition as “a belief… that there is a correct and desirable form of the language, distinct from normal practice” (Spolsky, 2004, p. 27). Language ideologies, being the primary driving force behind language policy, are rooted in the perceived value, power, and utility of languages. They are context-specific and intertwined with various factors including economic, political, socio-cultural, and linguistic elements, as well as parental educational experiences and expectations (Curdt-Christiansen, 2009). These factors, interrelated and potentially conflicting, can simultaneously influence individuals' belief systems and manifest in numerous social actions, and have significant implications (Gal and Irvine, 2019). This study considers the difference between the terms “language ideologies,” “language attitudes,” and “language beliefs,” and adopts Dołowy-Rybińska and Hornsby (2021) definitions. Language ideologies refer to socially shared beliefs, feelings, and assumptions regarding language, reflecting broader societal and cultural values. Language attitudes are explicit opinions shaped by these ideologies, influencing language choices and often socialized through various agents (e.g., family, teachers). Language beliefs are individual or collective opinions about specific languages or practices; they are not always socially shared or deeply ingrained.

Language ideologies extend beyond language use and encompass knowledge about various aspects of social life. They manifest in numerous social actions, sometimes in contradictory and contentious ways, and have significant implications (Gal and Irvine, 2019). Even in the absence of explicit language policies, implicit policies persist, reflecting collective beliefs about language use and standards.

Dismissing explicit rules or debunking the notion of “standard” languages does not eliminate the cultural assumptions ingrained in these concepts. The field of language ideologies explores people's “beliefs, feelings, and conceptions about language structure and use” (Kroskrity, 2010) rooted in sociocultural experiences, representing partial attempts to rationalize language use of the speakers. High awareness of a language role typically leads to active contestation of ideologies, while lower awareness tends to result in accepting dominant ideologies without question (Kroskrity, 2010). Language ideologies play a crucial role in shaping various social identities, including ethnic and national (Hoffman, 2007; Shankar, 2008), with shared languages historically defining social group boundaries (Kroskrity, 2010). Language ideologies are crucial for analyzing marginalized, converted, and appropriated identities (Kroskrity, 2010), with multiple studies illustrating how language ideologies shape acts of identity appropriation and hybridization, underscoring the role of language in social life (Kroskrity, 2016; Farella et al., 2021). Language ideologies are intertwined with processes of HL transmission, attrition and revitalization, being a crucial means for understanding multiple ideologies within multilingual communities, both in indigenous (McEwan-Fujita, 2010) and immigrant languages (King, 2000; Park, 2022). Within a family context, parental language ideologies, being shaped by their sociocultural and historical background as well as by the process of social integration into the receiving society (Curdt-Christiansen, 2009), assume a central role in HL vitality (Seo, 2021; Idaryani and Fidyati, 2022). Parental language ideologies are crucial in multilingual contexts as they shape the language environment and choices for children. They impact language policies, planning, and education within families (Zhang-Wu, 2024). Parents' beliefs regarding the value of different languages, their attitudes toward bilingualism or multilingualism, and their language practices at home significantly influence children's language development and identity formation (Cangelosi et al., 2024). To contribute to the current understanding of the language ideologies of multilingual transnational parents, this study examines individual parental beliefs and attitudes, identifying common patterns that represent the language ideologies within this specific linguistic and cultural community. While making generalizations based on the findings of a limited group of participants is challenging, certain trends remain significant.

### 2.2 Transnational multilingual families

Transnational families, defined by Cho et al. (2010), are families that live in two or more countries while maintaining close ties to their homeland and face similar challenges to immigrant families, but what sets them apart is the strong desire to preserve family connections. Some of the most notable and critical processes of transnationalism are arguably managing, learning, and maintaining their languages, mainly because the relocation experiences of transnational families diverge from those of traditional immigrant populations (Hirsch and Lee, 2018). The central negotiation in such families often revolves around the parents' HLs and the official language(s) of the receiving country, and with every new relocation and new language, the linguistic dynamics become increasingly complex. Balancing heritage and host country languages influences cultural preservation and assimilation—selecting the parents' HL fosters intergenerational continuity and maintains ties with extended family, while adopting the host country's language expedites assimilation. An additional option available exclusively in transnational families involves choosing one of the languages previously acquired by the family, which is neither the parents' HL nor the dominant language of the host country (Hirsch and Lee, 2018).

Braun and Cline (2014) introduce a notion of a trilingual family—a family where parents can offer their children two native home languages in addition to the community language, dividing such families into three major groups: (1) monolingual parents speaking different native languages and residing in a third language country; (2) at least one bilingual parent, with possible combinations of none, one or two shared native languages, either including or excluding the community language; and (3) either one or both parents are trilingual. The parents in the second group stand out for their conscious and deliberate language choices, often going against their emotional inclinations and prioritizing convenience and language status over personal preferences. The authors attribute a great value to parental attitudes toward minority languages, as well as to the cultural and social values associated with each language, as vital constituents in building the child's multilingual character. In a multilingual environment where people speak various languages at different levels, mixing codes is almost normal, even natural. As a consequence, the emphasis on language accuracy is much less than is the case for people who grew up with only one main language (Braun and Cline, 2014).

In transnational families, individual language ideologies can vary based on each member's perception of language status, influenced by global and immediate contexts. This article primarily discusses language status in terms of distinct languages within a specific community or society, briefly mentioning diglossia in terms of Low and High varieties of Russian, namely Israeli Russian and Modern Standard Russian (Spolsky, 2018). Language dynamics vary within families due to multiple moves, with siblings potentially having different dominant languages (Fogle and King, 2013), or previous languages being replaced by the dominant language(s) of the current setting (Haque, 2011). Family language ideologies may favor one language, while individual ideologies of the family members can greatly differ, influenced by personal experiences and interplay of factors (Haque, 2011; Phoenix and Faulstich Orellana, 2022; Kwon and Martínez-Álvarez, 2022). Irrespective of the context, the language ideologies that underpin language management and practices in multilingual families are intricate and frequently operate at an emotional level, but it is apparent that the experiences within transnational families undergoing various language learning and maintenance processes are diverse. These experiences surrounding transnational family relocations and separations significantly shape their ideologies and approaches to various aspects of life, including languages (Hirsch and Lee, 2018). Understanding these dynamics is crucial given the increasing prevalence of transnational families, necessitating consideration of time, plans, needs, and context during language negotiations, impacting family roles and ideologies.

The situation observed in the present study is unique—being born in dominantly Russian-speaking USSR, the participants were repatriated as minors to Israel, where they gradually shifted to Hebrew as their main language, but in a vast majority of cases retained communication and literacy skills in Russian (Niznik, 2011). As adults, they relocated to Finland, all the while maintaining close ties with their Russian-speaking families living in Israel. Consequently, they encountered another language choice alongside those previously discussed by Hirsch and Lee (2018): whether to preserve none, one, or both of their two native languages as HLs for their children.

### 2.3 Language ideologies in heritage language transmission

A developing body of research has explored the language ideologies and HL decisions among multilingual immigrants concerning their children, revealing diverse ideologies and varying degrees of family support of the HLs. In their book on parental perspectives in trilingual families, Braun and Cline (2014) devote considerable attention to situations where at least one parent is bilingual, and the family lives in a third-language environment. They provide examples from various language combinations in different countries and conclude that raising awareness about trilingualism will encourage parents and educators to see nurturing minority languages as beneficial for children's cognitive and social development (Braun and Cline, 2014). In Haque (2011) study of a plurilingual Indian family in Finland, the father's language ideologies, including the belief in irrelevance of Hindi and Urdu in Finland and high appreciation of Finnish, and the mother's ideology on maintaining the cultural identity through the HLs, hindered by her gradual shift toward Finnish and insufficient external support, lead to the decision for the children to be educated in English, with moderate support in Finnish. Warditz and Meir (2024) study illustrates how language ideologies of bilingual Russian-Ukrainian refugee parents toward language transmission in Europe underwent significant changes. Ukrainian gained higher symbolic status, while Russian retained a more favorable pragmatic role as a lingua franca in the diaspora, despite its negative symbolism. Nevertheless, in Austria, just over a quarter of respondents attributed importance to Russian for their children, while Ukrainian was favored in over 90% of cases. These studies demonstrate that bilingual parents' ideologies regarding HL choice for their children are influenced by a variety of factors, including perceived utility of the language, dominant languages of their environment, external support for HLs, and the geopolitical context. The present study aims to contribute to the growing body of literature on parental language ideologies in transnational families by exploring the language beliefs, attitudes and ideologies of bilingual Russian-Hebrew parents living in Finland.

### 2.4 Israeli Generation 1.5

The term “one-and-a-half generation” (Rumbaut and Rumbaut, 1976) refers to children and adolescents who have immigrated to a new country. They exhibit characteristics of both the first (immigrant) and second (native-born) generations, with slightly over half of them becoming balanced bilinguals—adopting Grosjean (1982, p. 223) definition, “those equally fluent in two languages”. They may exhibit a complex language usage pattern, potentially being dominant in either the heritage or the environment language, or identify with one language but actually have higher proficiency in the other one (Roberge, 2002).

Starting with the collapse of the USSR and till 2000, around a million people immigrated to Israel from the former Soviet republics (Perelmutter, 2018a). Most children of this wave, known as Generation 1.5, became “skewed bilinguals” within their first decade in Israel, primarily using Russian only with Russian monolinguals, while progressively shifting to Hebrew with younger family members, friends, and their own children (Remennick and Prashizky, 2019). For communication amongst themselves, they developed a code-mixed language formed as Russian under the influence of Hebrew, exhibiting lexical code-mixing, code-switching, phonological and morphological adaptation of Hebrew lexical elements into the Russian matrix (Naiditch, 2008; Perelmutter, 2018a), thus significantly differing for the Modern Standard Russian. Adopting Spolsky (2018) terminology, Israeli Russian is a Jewish variety used by Russian-speaking Jews both in Israel and in the diaspora. This perspective emphasizes that language use extends beyond mere communication to encompass cultural expression, identity negotiation, and social positioning. In the context of diglossia, various non-standard, so-called “impure” forms of Russian are considered Low languages (Bilaniuk, 1997). Israeli Russian, also taking into account the generally cautious attitude toward code-switching in the Russian-speaking Israeli community (Altman et al., 2014), also typically serves everyday language functions as a Low variety, while Modern Standard Russian is perceived as a High variety. Nevertheless, despite being frequently criticized by its speakers when discussed with outsiders, Israeli Russian still serves as a tool for strengthening community bonds (Perelmutter, 2018b).

Despite this shift away from Russian, there is recent evidence suggesting a growing tendency among Generation 1.5 individuals to rediscover the value of their Russian heritage and language, reclaiming their original identity (Prashizky, 2020). Nowadays, this large and relatively homogeneous demographic cohort in Israel, estimated to 170,000 individuals (~1.8% of the population of Israel), represents a relatively uniform group with a distinct, hybrid social, cultural and linguistic identity shaped by shared challenges (Remennick and Prashizky, 2023).

Determining the exact number of Israelis living abroad is highly challenging since there is a lack of direct data on the annual number of Israelis explicitly intending to emigrate. However, it is estimated that ~10% of Israel's population resides outside the country at any given moment (DellaPergola and Lustick, 2011). Data on emigration based on languages spoken is even more elusive, or even non-existent. In December 2022, Finland was home to 946 Israeli citizens, of whom 547 held dual Israeli-Finnish citizenship. Five hundred and eleven individuals identified as Hebrew speakers (Official Statistics Finland, 2023). It is crucial to note that Finland's language registration system allows the listing of only one mother tongue (Palviainen and Bergroth, 2018), which hampers the precise determination of the total number of Hebrew—another language bilinguals. According to a recent survey conducted on Hebrew-speaking parents in Finland (Bloch, 2024), out of 36 participants, 10 self-identified as Generation 1.5 Russian-Hebrew bilinguals.

## 3 Methodology and data

The present study is a qualitative case study examining language ideologies of bilingual Russian-Hebrew parents residing in Finland. The qualitative nature of the study can be observed via the method used; semi-structured interviews were chosen in order to obtain information on the participants' lived experiences that influenced their language ideologies toward language maintenance in their children.

### 3.1 Data collection

Participants were recruited via a survey on heritage Hebrew maintenance (Bloch, 2024), where the researcher reached out eligible candidates who had expressed interest in being interviewed for the subsequent research. Eligible candidates included adults who were repatriated to Israel from PSS as minors, later immigrated to Finland as adults, possessed native-level proficiency both in Hebrew and Russian, were parents with at least one minor child at the time of the interview, and were available for interviews within a month.

### 3.2 Participants

All participants in the study were aged between 40 and 50 years old and had two to three children, from 7 years old to young adults living separately (Table 1). The participants lived in various locations in Finland—in the Capital region (both one of the main cities and a small town), and in central Finland—both one of the major cities, and a small town with a population under 2000. One participant had recently moved to another European country after living in Finland for 20 years, marking the longest duration of residence, while the shortest residence period in Finland was 1 year. As minors, all participants had repatriated to Israel between the ages of 6 and 15, classifying them as Generation 1.5. The interviews were taken from both parents in case both of them were repatriated as minors (four participants, two families), and in three cases the participants' spouses moved to Israel from PSS as young adults, spoke advanced, but not native level Hebrew, and native Russian, and were not interviewed.

**Table 1:** Basic characteristics of the participants.

**Family**	**Research participant**	**Age of repatriation to Israel**	**Minor children^a^**	**Languages used with the children on a daily basis**
F1	Mom1	7	2 in primary school	Russian
	Dad1	9		Russian
F2	Mom2	9	1 in JH school, 1 in primary school	Hebrew, Finnish
	Dad2	7		Hebrew
F3	Dad3	15	2 in JH school, 1 in primary school	Russian
F4	Mom4	15	1 in JH school	Russian
F5	Mom5	15	1 in high school, 1 in JH school	Hebrew

^a^Information about adult children living separately is not included in the study and is therefore not mentioned here.

Strict adherence to GDPR ensured participants' privacy and data protection. Participants were fully informed about the study's purpose, data handling, and their rights, including the right to withdraw or request to delete interview parts, and provided written formal consent. Confidentiality and anonymity were prioritized throughout the research, with all potentially identifying information removed or generalized in the findings.

This study acknowledges several limitations regarding the participants. Despite the complexity and diversity observed among the participants, the paper's findings are based on a limited sample size, thus not claiming generalizability to other multilingual transnational families and contexts due to its small scale. The study's reliance on participants willing to engage may introduce selection bias, offering only a partial perspective on the phenomena.

### 3.3 Interviews

The interviews served as a continuation of the research on Family Language Policies of Hebrew-speaking parents in Finland (Bloch, 2024). The semi-structured interviews comprised the topics of parental language ideologies, linguistic management within the families, actual linguistic practices, and languages as factors influencing the children's social inclusion in Finland. The interviews were conducted through April–May 2024, either in person or via University of Helsinki Zoom, and the recorded parts lasted ~1 h per participant. The interviews with families where both parents were interviewed were conducted as follows: each parent was interviewed separately first, with the second spouse either absent or present based on their own preference. Following the individual interviews, both parents were interviewed together for clarifications, a process initiated by the families themselves.

To examine the interviewees' attitudes toward and ideologies concerning the languages they are exposed to, namely, Russian, Hebrew, Finnish (although Finland is officially a bilingual country, no participants resided in Swedish-speaking regions), and English, as well as other languages, such as the languages of PSS the participants repatriated from as minors, semi-structured interviews were applied as a research tool to facilitate flexibility and free and open conversation. Each interview was structured according to the following themes: (1) background questions (2) linguistic practices in the family (3) explicit language policies (4) ideologies toward the languages used in the participant's family, as well as general attitudes toward multilingualism (5) language management (6) emotional aspect of the participants acquiring Hebrew as minors, and their feelings toward their children's language acquisition in different stages of life (7) external factors influencing parental decisions on language choices for communication with the children (8) connection between the children's language acquisition and their social inclusion. The interviews were part of a broader project on multilingual Israeli families in Finland, and in the current article, in addition to considering the participants' backgrounds, the focus was primarily on their language ideologies. The study considered both linguistic and social structures, documenting different patterns of language use as well as relating these findings to the wider social context (Soler and Zabrodskaja, 2017).

The interviews flowed between languages, mostly in Israeli Russian—code-switching between Russian and Hebrew, with insertions in Finnish and English. Participants were encouraged to use the language(s) in which they felt most comfortable and natural, being aware that the researcher was also a Russian-Hebrew bilingual residing in Finland. Overall, 7.5 h of interviews were recorded, with an average of ~1 h per participant. The interviews were transcribed, and parts of them were translated into English.

### 3.4 Data analysis

Thematic analysis was implemented to identify patterns in the participants' responses. The analysis followed Kroskrity (2010) approach to language ideologies, which underscores their multifaceted and contested nature. This approach shows that language ideologies are neither uniform nor entirely distinct; rather, they can be explicitly articulated or implicitly embedded in people's actual language use. Additionally, it emphasizes the significance of comprehending the variation and diversity of language ideologies within cultural groups. Thematic analysis revealed recurring language ideologies, which aligned with those documented in various studies on language ideologies within multilingual communities. Thematic analysis was performed using Atlas.ti software to discern patterns in the participants' responses, ultimately leading to the categorization of the language ideologies articulated by the participants. The six main areas of application of the ideologies were coded according to the four languages in question—Russian, Hebrew, Finnish, and English, as well as multilingualism in general and code-switching. The recurring language ideologies in every area of application were identified according to the theoretical frameworks used in analyzing language ideologies within multilingual transnational environments across various scholarly works and publications.

## 4 Findings

This section provides an overview of key themes identified through semi-structured interviews with the seven participants. Beginning with the description of general ideologies toward multilingualism, the section proceeds into the ideologies underlying language choices for communication with the children, code-switching, and reflections on the correlation between language proficiency and social inclusion in Finland.

### 4.1 Ideologies concerning multilingualism

Transnationalism typically involves the processes of language learning, acquisition, and maintenance or loss. It encompasses the development and maintenance of language skills needed to establish and sustain complex multinational and multilingual relationships in transnational families (Hirsch and Lee, 2018). Prior research identifies the family as a key context for exploring ideologies, management, and practices of various languages among different family members (Spolsky, 2012), with parental ideologies shaping children's identity, self-esteem, and academic success (Lee and Suarez, 2009). This section explores explicit comments about language choices, as well as recurring beliefs, attitudes, and practices found in the interview data.

#### 4.1.1 Multilingual advantage

Throughout the interviews, a notable appreciation for multilingualism emerged, reflecting a common parental belief underlying the ideology that multilingualism is the most natural and beneficial language practice for their children. To support this multilingual advantage ideology, parents provided various explanations, with the concept of functional value being the most frequently mentioned.

(1) Every language is like another key, a global advantage. In the future, they will be able to find contact with people from different regions or from different countries. They will be able to develop themselves, develop their career and family anywhere. And they will be able to use these keys, because every language is like a supplementary item that can be easily launched to cope with the tasks that he has in every new place (Dad2).
(2) In general, they have a broader view of the world in a global sense. When one is constrained by one language, one seems to think inside this linguistic layer, and does not see what is outside. I see how my children they feel in any country they go to, they feel much freer than me or my mother, not to mention generations before. Theoretically speaking, soon everyone should … move everywhere without problems and speak all the languages they want (Mom4).

#### 4.1.2 Freedom of language choice

Concerning language choices made by the children in Finland, a notable pattern emerged: all the parents emphasized the freedom of choice and parental support of this choice:

(3) If my kid comes to me and says—I live in Finland, and from now on I speak only Finnish, I'll say—it's your choice (Mom4).

Enabling free choice presents a challenge for many migrant parents (Lizardo, 2017), especially those from the PSS, who typically instill their children with clearly defined values and adhere to rigorous methods (Leybenson et al., 2023). While participants commonly expressed support for their children's freedom in language choices, instances of parental intervention arose when the children's choices conflicted significantly with parental linguistic ideologies. Examples 4 and 5 describe such instances for Russian and Hebrew, respectively:

(4) For selfish reasons, we decided that we would support Russian at home at any cost and we stuck to this. In particular, when the children were smaller and when there was a period of shame for the Russian language and when their Finnish friends came to visit, they hissed at me, like mom, don't speak Russian to me, well, I kind of stuck to the line that at home we speak Russian. Of course, I will speak Finnish with your friends. But with you Russian. That is, they made attempts to sort of switch to Finnish at some point, even in homeschooling, which we put an end to. These are the decisions that were made (Mom4).
(5) Dad should be called dad in Hebrew, and dad will answer in Hebrew, and they won't wait for an answer in Finnish, because they know that Finnish is, well, not the language we communicate in (Dad2).

The examples above illustrate parental preferences when opposing ideologies intersect. While all participants discussed the ideology of language choice freedom, this freedom is often overridden by ideologies of personal convenience (4) or cultural value (5), revealing a hierarchy of language ideologies.

### 4.2 Parental language ideologies

All participants, aged between 40 and 50 during the interviews, were born in the USSR and immigrated to Israel as school-age minors. They initially attended Russian-medium schools for 1–9 years before transitioning to Hebrew-medium schools, while Russian continued to be maintained at home. It is essential to consider an external language ideology of language superiority in Israel during the participants' childhood, as they influenced their own linguistic ideologies. Parental justifications underlying their language choices in Israel revealed a number of recurring patterns, shaping a number of distinct language ideologies within the participant group.

#### 4.2.1 Language superiority

This ideology at that time concerned both languages—Russian and Hebrew, with each language claiming to have superiority over other languages. The ideology concerning the superiority of Russian, leading to strong encouragement to speak Russian at home in Israel, significantly contributed to the participants' continued use of Russian during childhood. This encouragement was reported unanimously by all participants, leading to Russian becoming the primary or sole home language for most. This reflects a common trend in Israel, where many Russian-speaking immigrants prioritize preserving their language and culture for the next generation, often considering Russian superior to Hebrew (Niznik, 2011). They actively encourage their children, including those born in Israel, to learn and use Russian (Schwartz et al., 2009). A recent survey of Russian-speaking mothers across four countries found that 96% of respondents in Israel reported that their children could speak and understand Russian (Otwinowska et al., 2021). Nevertheless, despite the parents' consistent efforts to maintain Russian as the home language in Israel, one participant reported choosing to forego Russian during childhood, referring to the ideology of language superiority regarding Hebrew—the rarely contested Hebrew-only national ideology that endured until the end of the 20th century (Helman, 2002). The ideological dominance of Hebrew monolingualism led most immigrants to prioritize Hebrew over their home languages, resulting in subtractive rather than additive bilingualism within the next generation, and a gradual shift away from home languages. The attitude toward multilingualism began to change in the mid-1990s, as reflected in the Ministry of Education's establishment of a Language Education Policy that endorsed multilingualism (Donitsa-Schmidt, 1999). One of the participants repatriated to Israel at the age of 7 just before the collapse of the USSR attributed his personal experience of subtractive bilingualism to the prevailing Hebrew-only national ideology:

(6) My mother spoke to me in Russian, I heard Russian speech, but I answered her in Hebrew. I had some kind of restrictions or insecurities, one might say, related to Hebrew and the fact that my mother did not know Hebrew well. And at school they said—“Speak Hebrew at home with your parents. This way, they will learn Hebrew faster.” This was their policy. Or they insisted and through us they endured this kind of pressure on parents to improve their Hebrew language. I can simply say that I always had the Russian language in my head, but I didn't use it. … When at the age of 16 I got Russian-speaking friends, and they did not know any other language, I had to speak Russian, and I restored the Russian language. You see, it was always there, it never disappeared (Dad2).

All the respondents had hybrid-culture, Russian-speaking Israeli partners. In three cases, the participants' partners had moved to Israel as young adults (aged 18–22) and acquired Hebrew at an advanced, but not native, level, while the rest were representatives of Generation 1.5 of the 1990s ex-Soviet immigrant wave. This tendency aligned with Remennick and Prashizky (2019) findings from longitudinal ethnographic studies, which examined the social, cultural, and political positions of young Israeli adults from Generation 1.5, where 70% of the 650 respondents had similar partners.

#### 4.2.2 Personal convenience

All the participants cited personal convenience as a valid theoretical justification for HL choices. Some identified Russian as more convenient, others preferred Hebrew, and some found both languages equally convenient. Examples 7 and 8 illustrate the choice of the HL based on personal convenience:

(7) It seems that despite the fact that my husband and I speak Russian, and it is kind of a native language from home, and we both speak Russian well, but we always spoke Hebrew between us. For us, it was like our common language, and when the eldest was born, we kind of did the same, we continued communicating in Hebrew (Mom2).
(8) I set myself a goal that I want to speak Russian at home with my children. This is a purely egoistic desire, in fact, behind which there is nothing deeply philosophical; I wanted to at least be able to maintain closeness with them, because first of all, I can best express myself in my own language (Mom4).

The choice of Hebrew, in additional to personal convenience, was also manifested via a combination of factors, beliefs, attitudes and practices that repeat in the data, such as the self-assessment of Hebrew as the participants' strongest language—(9) “my main language is Hebrew, and I am completely literate only in Hebrew” (Dad2), (10) “I just switched to Hebrew so that the children could hear good, correct Hebrew” (Mom5), (11) “[Hebrew] is my native language. It's easiest for me to express myself and communicate in Hebrew” (Mom2). The desire to transmit both HLs was mostly based on the common ideology of multilingual advantage; nevertheless, only one participant decided to stick to the OPOL “one parent—one language” method, where each parent consistently speaks a different language to their child, an approach aimed to create a clear linguistic environment that supports bilingual development (Barron-Hauwaert, 2004). Other parents showed regret they were unable to do so, mentioning personal lack of time, skills, which was consistent with my previous study (Bloch, 2024), and which emphasized the ideology of parental responsibility for HL maintenance.

(12) I regret that we didn't keep 2 languages in the family, we didn't speak one [parent] in Hebrew, one in Russian. I really regret this (Mom1).

#### 4.2.3 Cultural and traditional value

Participants discussed the cultural value of each HL, mentioning literary heritage in both languages, as well as traditional values specifically linked to Hebrew. Interestingly, when considering the possibility for their children to read the most prominent literary works in the original languages, participants expressed doubt about their children reaching this level of proficiency. This acknowledgment underscores their aspirations and simultaneously recognizes the challenges involved in transmitting cultural values associated with literary heritage.

(13) Most likely they will not read Pushkin in the original. But then again, life is full of surprises—it could happen. Maybe they will, and I will be endlessly happy (Mom2).
(14) Well, their today's Hebrew is far away from Bialik (Mom5).

The belief of the importance of Hebrew as a means of maintaining the Jewish traditions for the cultural continuity was discussed by one parent:

(15) In our house, Judaism stands at a fairly high level. Not the religion itself, but the tradition, elements of the tradition, they are at a fairly high level. We have this as a priority, and we think that language is part of keeping traditions, because everything is written in Hebrew and everything is explained in Hebrew (Dad2).

Case studies in Jewish communities in the diaspora reveal a strong connection between Hebrew and religion as well as Jewish values. For instance, interviewees attending American Jewish Summer Camps shared similar sentiments, asserting that Jewish texts must be accessed in Hebrew (Benor et al., 2020).

#### 4.2.4 Parental responsibility

The ideology of parental responsibility for HL maintenance was born form the strongly positive attitude toward multilingualism and the desire to raise bilingual children. The future language strategies of the present study participants were either pre-discussed before having children or evolved naturally, depending on the languages used between the partners. However, the primary determining factor was their residence in Israel at that moment, with no plans or discussions regarding relocation to a third country, making them bilingual parents with two shared native languages, including the community language. The ideology of parental responsibility lead some of the participants to choose Russian despite feeling more convenient in Hebrew and recognizing the imperfect knowledge of Russian:

(16) We only discussed this in Israel when we were deciding what language we would speak at home. Yes, we wanted to, because you know, at 3–4 years old children go to a municipal kindergarten and that's it, the additional language disappears, so we specially sent him to a Russian-speaking kindergarten so that the child could learn Russian, to give them him an additional language (Mom1).

While various case studies suggest that bilingual parents tend to opt for the language they believe they are more proficient in (e.g., Braun and Cline, 2014; Chiswick and Gindelsky, 2016), the choice of HL between Russian and Hebrew did not exhibit a clear correlation with participants' self-assessed language proficiency, age of repatriation, or duration of attendance at Russian- or Hebrew-medium schools. Among the three participants who were repatriated at age 15, two chose Russian mostly for personal convenience, while one selected Hebrew to maintain the “one parent–one language” (OPOL) strategy with their children, whereas of the two couples who repatriated between ages 7 and 9, one opted for Hebrew, and the other for Russian, despite acknowledging that Hebrew was their stronger language:

(17) I can write Russian correctly and speak it kind of correctly. Well, sometimes I don't speak it correctly, because I haven't studied it formally anywhere, that is, I know Russian only from what I've heard. And I read, but I write probably with some mistakes, but again I didn't study at school, I didn't study in Russian, and I didn't study Russian as a subject anywhere (Dad1).

Dad4 and Mom1 shared concrete plans for his children's Hebrew maintenance for the nearest future in case they decide to learn Hebrew, namely, sending them to live at the grandparents' and registering them in a local school for a few months. These examples represent an intersection of two mutually supportive ideologies—freedom of language choices for the children and parental responsibility.

In contrast to the efforts put into maintaining Russian and/or Hebrew, parents entrusted the responsibility of teaching English to their children to the school system. They expressed confidence in the Finnish educational system's ability to provide their children with a solid English education. As Dad1 put it (18), “English will come naturally through school,” while Mom1 stated (19), “they will not know it as a native language, but it seems to me that they will know it quite well, since I can see what kind of system there is.”

In line with prior research findings indicating the significance of education and scholarships as fundamental values and primary socialization goals for 1.5 generation PSS Israeli parents, and their prevailing belief that parents bear the responsibility of dedicating significant time in teaching their children (Ulitsa et al., 2020), most research participants emphasized their own duty to transmit the HLs to their children. They regarded external HL support as secondary, and even unnecessary, and assumed full responsibility for the children's language attrition.

Regarding institutional HL support in Finland, children from immigrant backgrounds, extending to the third generation, have the right to receive 2 h of additional basic education in their “mother tongue” or “home language” (“oma äidinkieli” or “kotikieli”) aimed to promote active multilingualism, with an emphasis on enhancing native language literacy and developing multiliteracy skills (OPH, 2022). Two participants reported enrolling their children in home language classes organized by the municipalities, but were dissatisfied with the quality of teaching and even mentioned traumatizing experiences for their children. Regarding Hebrew education, several participants attempted Hebrew lessons, but none were satisfied with the process:

(20) Hebrew was taught horrendously incompetently and the auntie who taught it was not a teacher at all, but just some kind of an Israeli auntie who, for some reason, decided that she could actually teach the language just for the fact she was Israeli, so she taught it horrendously (Mom4).

One participant enrolled their children in municipal Russian classes and recounted the trauma it inflicted, which significantly impacted their children's attitudes toward Russian literacy for years. As the participant expressed (21), “the terrible [teacher's name] instilled an aversion to learning the Russian language in my eldest child for the rest of her life.” Over half of the parents either were unaware of the opportunity to enroll their children in HL classes or admitted to disregarding proposals from schools. One participant initially couldn't recall being offered HL classes during the interview but later recollected, after consulting with her children, that Russian as a HL class had indeed been suggested by the homeroom teacher. However, she acknowledged not paying enough attention to this proposal at the time.

To summarize, the findings of the study aligned with previous research indicating the profound importance of education and scholarships as core values among Generation 1.5 Post-Soviet Israeli parents (Remennick, 2003; Niznik, 2011; Schwartz et al., 2009). Most participants emphasized their responsibility to transmit HLs to their children, prioritizing parental efforts over external language support and assuming full accountability for their children's language development. Concerning home language classes provided at the state level, some participants were not aware of this opportunity, while others encountered difficulties with HL classes organized by municipalities, which led to dissatisfaction and reported traumatic experiences for their children. All the participants trusted the Finnish education system for Finnish and English instruction.

#### 4.2.5 Functional value

While some parents considered it essential to use the language they feel more convenient in to be the language they use with the children, for others the personal belief and the societal attitude to multilingualism coupled with own desire to raise bilingual children lead them to giving up to personal convenience and using the language they feel less connected to. This parental decision to use Russian in this case aligns with Braun and Cline (2014) finding that bilingual parents often make conscious and deliberate language choices, prioritizing the functional value of a language over personal preferences, even if it goes against their emotional inclinations. Dad1 decided to transmit Russian as the main HL to the children because (22) “Russian is the only language that everyone [in the extended family] knows,” a choice supported by his spouse Mom1, who communicated only in Russian with older relatives in Israel. Both participants emphasized that the decision to use Russian was made before the family's relocation to Finland.

While all the participants expressed overwhelming positive attitudes toward multilingualism, in most cases it went independently of any specific languages, except English. The more languages on a good level—the better was the leitmotif in all the interviews, and English emerged as holding great significance in all the interviews, with its functional value as a lingua franca for more extended communication, or as one of the main skills for educational purposes, gaining such explanations as (23) “a universal language that reached the goal of Esperanto,” (24) “with English any international opportunities are open.” Considering English the most useful language for the children also fits into the ideology of functional value. This perspective aims to transform the children's English language skills into symbolic capital, which can provide social and economic advantages in the future (Woolard, 2020). In line with previous case studies, where immigrant parents considered English as a more important language for the children than the language of the environment (e.g., Cangelosi et al., 2024; Dołowy-Rybińska and Hornsby, 2021), a similar ideology was voiced—all the participants mentioned English as the most useful and practical language for the children's communication opportunities, studies and future career:

(25) Because if they learn English well, they will be able to communicate with a lot of people, and then Russian and other languages will not be entirely important (Dad1).

The responsibility for attaining English skills was attributed mostly to external sources, such as school and communication with international friends, and at home was used for support with studies, for instance, learning scientific terms in physics. Most participants praised the level of English teaching in Finland, attributing to it their children's high level of English, while one participant reported that all her children learned English on their own and mostly from TV. One participant expanded on their decision to enroll the children to an English-medium instruction school:

(26) We decided that English is obvious, that is, they can find it anywhere, so to some extent we put English, well, on a par with Russian. That is, Russian is the language of the family, and English is the language of the outside (Dad3).

Finnish was not commonly spoken within the participants' families on a daily basis; nevertheless, its significance and value for the children emerged prominently in most interviews. Reasons cited included personal fondness for the language due to its perceived beauty, future aspirations for the children such as attending a Finnish university, recognition of Finnish as vital for successful integration, and the aspiration for the children to acquire proficiency in multiple languages. Finnish was widely regarded as a valuable skill by most participants. In addition to being the primary language of the environment for all but one participant's children, it was supported for educational purposes by all the families. Even participants with weak Finnish skills took the initiative, without instruction from school, to implement home practices, such as requiring their children to read aloud in Finnish for 30 min or translate scientific terms from English textbooks into Finnish. Ultimately, all participants believed in the school's willingness and ability to support their children's Finnish language development.

Overall, the experiences of the study participants highlighted the complex dynamics of HL maintenance and adaptation to the new linguistic environment. Their language choices were influenced by various factors, including personal convenience, cultural ideologies, and practical considerations, but they all underscored the importance of preserving HL(s), either one of them or both, while adapting to life in Finland. The participants' narratives illustrated the dynamic interplay between HL preservation, adaptation to new linguistic environments, and the strategic incorporation of English. This interplay was characterized by a nuanced understanding of the benefits of multilingualism, the importance of cultural continuity, and the functional value of raising children in a multilingual world.

### 4.3 Ideologies concerning code-switching

In line with Remennick's sociological study (2003), where 58% of 1,000 Generation 1.5 representatives reported some degree of code-switching between Hebrew and Russian, most participants in this research acknowledged engaging in code-switching to some extent. Besides deliberate code-switching, subtle markers such as occasional insertions of Hebrew or English terms in otherwise fluent Russian, like the Hebrew/English calque “carrot” from the “carrot and stick” proverb, meaning “whip and sugar bread” in Russian, or sporadic errors in noun case forms typical for Israeli Russian (Meir et al., 2021), were observed. Participants reflected on their code-switching and language mixing behaviors, providing examples of situations where they switch between languages or specific words they use exclusively in one language.

(27) I have one word that I can't get rid of, it's gina [Heb. “garden”], it's not piha [Fin. “garden”] for me at all, I even tell my children that it's not piha for us, it's gina (Dad3).
(28) Between us [the spouses], not really, we don't speak Russian. Well, if some words are sometimes peculiarities of languages, when some word is, like, exactly, what I can say only in Russian (Mom2).

The two examples above illustrate the ideology of personal convenience, with most participants mentioning their own code-switching practices as a means of speeding up the communication or complementing the vocabulary in one of the languages with the words from another, either when the counterpart is too long or complicated, or when the word in the other language is non-existent: Dad3 gave a list of army positions in Hebrew that he uses in Russian, while other participants discussed the words in Russian which exact counterparts do not exist in Hebrew, but appear to be useful in Finland, such as *moroška* [Rus. “cloudberry”] or *izmoroz'* [Rus. “rime”, “hoarfrost”].

However, attitudes to code-switching and language mixing varied drastically. While for most participants inserting certain words from other languages was openly reported as “inevitable”, “no negative feelings, I know where it comes from”, or even openly supporting the code-mixed Israeli Russian for personal use, with Israeli Russian performing the function of a “tool for intra-communal affiliation” (Perelmutter, 2018b, p. 136):

(29) When we understand that around us [in a situation where Russian is lingua franca] there are people who also understand Hebrew, then there is some kind of relaxation, and an additional language appears. From my point of view, this is just incredibly cool (Mom1).

Nevertheless, all the participants reported continuously correcting code-switching instances within their children (30) “I take it with humor, but correct it” (Mom2), with all of them using one of the two practices—either asking the child what the code-mixed phrase be in one language (31) Dad3: “How would you say it in Russian?”, or repeating the phrase after the child, but in one language:

(32) I translate the whole thing back to Hebrew for them, and they look at me and repeat after me (Dad2).
(33) I repeat in Russian, when they, for example, ask, when is dad's palkka [Fin. “salary”]? I say dad's salary is on the 5th. That is, I try to duplicate it in Russian, but intentionally I don't put special emphasis on it (Mom4).

The practice of code-switching correcting within all the participants indicates on the presence of the ideology of multilingual disadvantage, which aligns with both the wide-spread general ideology on code-switching as negative, a sign of language decay and assuming that it negatively effects the proficiency in one or both languages (Aitchison, 1991), as well as with the local ideology that using Hebrew loanwords in Russian is a sign of HL Russian decay (Kopeliovich, 2010). Consistent with this, one participant expressed concern that Israeli Russian could become the main language for the children, and another one expressed openly negative attitudes toward code-switching in any languages:

(34) Even my relatives also had and still have this problem—they mix up languages, insert a lot of words from Russian into Hebrew, from Hebrew into Russian, and it turns out to be some kind of a salad. One needs to create some kind of division; in some way one just needs to limit themself in this and force themself to speak purely in the language that one communicates at the moment. This salad—I understand where it comes from, but as for me, it's wrong, it's distorting the beauty. There are people who are friends with this, but I think that this is a big drawback. If you speak, let's say, Russian, you must adhere to this (Dad2).

Within the framework of diglossia, this attitude reflects the perception of any non-standard variety of Russian as a Low language (Bilaniuk, 1997), and Israeli Russian in particular is frequently criticized by its speakers when mentioned to people from other backgrounds (Perelmutter, 2018b). Although the participant was interviewed by a community member, it was evident to him that the conversation was intended for an audience outside the community.

The attitudes toward code-switching differed depending on whether it was insertions of words in one language to another, or switching between languages in full sentences for practical purposes, such as for not being understood by others, i.e., secret language. The second case was perceived positively or even encouraged by the parents, who considered it a teaching opportunity to show the children additional advantages of multilingualism.

### 4.4 Languages and integration

Most participants discussed the correlation between their children's proficiency in Finnish and social integration into Finnish society, revealing a distinct division among parental ideologies. While some parents viewed mastering Finnish as essential for successful integration, others doubted that even native fluency would ensure complete integration. Conversely, a third group posited that integration in Finland could occur independently of proficiency in Finnish. Mom1, who had resided in Finland for the shortest duration among the participants, observed a direct link between her children's level of Finnish proficiency and their successful integration. She noted that, while both her younger children spoke English, her middle daughter quickly grasped Finnish, resulting in her having “an unbelievable number of Finnish friends” and feeling as though she had grown up there. In contrast, the youngest daughter struggled with Finnish, making it challenging for her to make friends.

Participants supporting the notion that children with an immigrant background inevitably struggle to fully integrate into Finnish society, pointed to two main factors. Some mentioned the creation of language bubbles within immigrant groups, while others highlighted the practice of segregating children with immigrant backgrounds in schools.

(35) Even if the kids will speak native Finnish, they will still be in the immigrant bubble and it's a no-nonsense bubble, but I mean. There are two separate worlds, no matter what, even if they were born here. Of course, it certainly depends on them how open they will be and how far they will have to leave their comfort zone in order, so to speak, to increase the circle of communication, but from what is naturally happening, what I see is that everyone with whom the children communicate is either the children of immigrants who came here, that is, the children themselves are immigrants, or even if the children were born here, their parents immigrated, and they've been living here for 20 years (Dad3).

Mom4, who had resided in Finland for 20 years, recounted several instances where her youngest child, born in Finland and fluent in Finnish, was forced to additional Finnish classes for children with immigrant backgrounds. After parental intervention, teachers confirmed the child's native-level proficiency in Finnish, acknowledging that the placement was solely due to the child's background. Mom4 described another instance of what she termed “classical segregation” her child experienced:

(36) There was an unpleasant incident when there was a celebration of 100 years of Finnish independence, this famous one, when these unfortunate children [with an immigrant background] were taken to matinees separately as trained monkeys, and they had to tell why they liked life in Finland so much. And my child, and several other children who had this background, but at the same time they did not live anywhere except Finland, they were born there, they spoke Finnish to each other—they could not answer this question—why they like living in Finland. As if they had another country to compare with. And that, in my opinion, was a pretty disgusting show (Mom4).

Another participant viewed successful integration as a process not solely dependent on mastering Finnish. They argued that since English was widely spoken by the majority of residents in Finland, proficiency in English could also contribute to successful integration. Additionally, they suggested that integration into the Russian-speaking community in Finland could also be considered as a form of successful integration.

(37) Firstly, there are a lot of Russian speakers here, so you can very easily find activities for children and communicate with Russian-speaking children. There are a lot of children here, and people in general, speaking English. This makes integration and everyday life in general much easier. With Finnish it is much more difficult, because since such an opportunity to communicate in both native and familiar languages, it greatly interferes with communication in the local language (Mom1).

To summarize the key findings, the participants' ideologies regarding language transmission, multilingualism, code-switching, and integration into Finnish society reflect a complex interplay of personal experiences, cultural values, and practical considerations. The attitudes toward language choices and practices showed diverse patterns, with participants choosing Russian, Hebrew, or both languages for transmission to the children in Finland. The most prominent language ideologies concerned the choice between personal convenience and functional value, with the latter influenced by factors such as the possibility of communication with all family members, integration, or educational goals. Code-switching was perceived as natural among participants, with attitudes ranging from acceptance to concern. Both English and Finnish held high functional value, with English holding more significance for its practicality and educational benefits. Regarding integration into Finnish society, participants held differing views. While some believed that proficiency in Finnish was essential for successful integration, others questioned its sufficiency.

## 5 Discussion

The primary objective of this study was to investigate the language choices reported by the seven participants, and the language ideologies linked with these choices. Transnational multilingual families offered rich grounds for exploring the complexities of managing linguistic resources and ideologies within micro-level contexts. Language-related decisions in these families were deeply integrated with all aspects of child rearing, impacting every family member (Soler and Zabrodskaja, 2017).

This study examined the language ideologies of transnational bilingual parents residing in a third language environment, with a particular focus on their language choices and policies upon relocating to Finland. While the participants strongly advocated for multilingualism, a key finding emerged regarding their consistent language choices upon relocating to Finland. Despite reflecting and discussing future language policies for their children while in Israel, the participants did not alter or reassess these policies upon moving to Finland. The policies typically revolved around raising children with two strong native languages, with Russian as the home/heritage language, or as native speakers of Hebrew with some heritage Russian background. Notably, only one participant continued to employ the OPOL strategy in Finland, while others, who had not considered it previously, did not adopt it. This finding aligned with Braun and Cline (2014) assertion regarding the challenges of implementing the OPOL strategy in trilingual settings, often leading bilingual parents to neglect one of their native languages.

Another intriguing finding was that many participants did not have a predetermined set of languages they wished their children to master. While they expressed a desire for their children to speak definite languages, such as Hebrew or Russian, but also emphasized the importance of their children's autonomy in selecting which languages to master. Their primary concern was not the specific languages spoken but rather that their children excel in at least one language, ideally several. This ideology reflects the power dynamics at play, the ability to switch between languages being a form of linguistic capital that can be leveraged in different social contexts (Schieffelin et al., 1998). The most significant worry among parents regarding multilingualism was the prospect of their children lacking full proficiency in any language, thus necessitating frequent code-switching to convey their thoughts. This finding went in line with Ballinger et al. (2020) research findings, where HL parents expressed more worry about the potential negative consequences of childhood multilingualism compared to non-HL parents. While the current research participants were concerned mostly about language mixing, Ballinger et al. (2020) also discussed overloading the children with too many languages, and potential delays in language development—the disadvantages not mentioned by the present study participants. Another point connected to language choices was not reported by the participants to play a role in HL choices—language status as an influencing factor. While bilingual parents in a third language environment often emphasize practical considerations and language status rather than personal preferences (Braun and Cline, 2014), the participants in this study primarily based their decisions on personal convenience, with the children's language preferences being a secondary consideration.

Conflicting language ideologies can exist even in small-scale societies (Woolard, 2020). The participants' attitudes toward code-switching varied across different contexts: their own practices in Israel, their current behaviors in Finland, and the code-switching observed in their children. In Israel, all participants reported engaging in code-switching, which was perceived as a norm within the community. This finding aligned with previous research on Israeli Russian (Naiditch, 2008; Perelmutter, 2018a). However, upon relocating to Finland, most participants' ideologies on code-switching underwent a shift. They made deliberate choices to use one language without incorporating elements from the other, citing reasons such as the desire to transmit at least one HL in its standardized form and the need to ensure mutual understanding when communicating with monolingual individuals, predominantly from Russian-speaking countries and occasionally from Israel. On the other hand, over half of the participants felt a sense of belonging, relaxation, and enjoyment when conversing in Israeli Russian with peers. Indeed, the sense of belonging to the Jewish community in the diaspora has always been represented through distinctive features such as clothing, foods, and language, with the differences in language characteristics ranged from being largely unintelligible to other speakers to including just a few added Hebrew words (Hary and Wein, 2013). This phenomenon is illustrated in Example 27, where the participant highlights the significance of using a Hebrew word to describe something as personal and relaxing as their own garden.

In regard to their children's code-switching, participants contextualized this phenomenon based on their own childhood experiences and considered it a natural aspect of language development. However, they consistently reported correcting code-switching in their child's speech by repeating the phrase in one language or asking the child to repeat the phrase. This practice employed by the participants in the study to correct their children's code-switching represents an example of the ideology of multilingual disadvantage, corresponds to a broader language ideology on code-switching as a sign of language deterioration (Aitchison, 1991) and decay the HL (Kopeliovich, 2010). Ideologies carry moral and political weight because they suggest not only how language is but how it should be. They assign more value to certain linguistic features or varieties over others, depending on the situation and the speaker. This can turn some people's language practices into symbolic capital, leading to social and economic rewards (Woolard, 2020). Consistent with this, some participants linked the higher value attributed to “correct” Russian, as opposed to code-switching Israeli Russian, to their children's future ability to create valuable connections with Russian speakers worldwide.

Regarding collective parental ideologies toward supporting HLs in their children, all parents viewed HL maintenance, whether Hebrew or Russian, as their personal responsibility, often forgoing or neglecting external support available to immigrant children in Finland. They emphasized the importance of oral proficiency in at least one HL, prioritizing this aspect over literacy, which was seen as beneficial but not essential.

Finnish, being the dominant language of the environment, held varying degrees of importance among the participants, ranging from a preference for their children to primarily use Finnish in their future endeavors to a recognition of its significance while residing in the country. Consequently, formal education languages ranged from strict Finnish instruction to bilingual English-Finnish programs, with a stronger emphasis on English in some cases. English emerged unanimously as a vital life skill for the children, with the responsibility for teaching it primarily falling on the school system. Parents generally expressed satisfaction with the level of English instruction provided in Finnish public schools. Furthermore, some participants highlighted the functional value of English for their adolescent children, aligning with previous research indicating that English has become a secondary language in the linguistic repertoire of Finnish adolescents (Leppänen and Nikula, 2007).

Parental ideologies on their children's proficiency in Finnish and its correlation with social integration into Finnish society revealed a nuanced spectrum of parental ideologies. Successful integration of immigrants, as outlined in a press release by the Ministry of Economic Affairs and Employment of Finland, involves multiple dimensions, including effective communication in Finnish or Swedish, employment, social participation, and societal receptiveness, with engagement needed from both the receiving society and the immigrants themselves (Ministry of Economic Affairs Employment, 2020). Some parents firmly believed that mastering Finnish was essential for successful integration, while others expressed doubts about the extent to which native fluency in Finnish could ensure complete integration, with a third group positing that integration in Finland could occur independently of proficiency in Finnish. Finnish integration policy emphasizes that learning the language and adopting cultural norms helps migrant children integrate into the labor market and contribute to society, with programs designed under the presumption that migrants are often disadvantaged or underprivileged (Zacheus et al., 2019; Korpela, 2023). This presumption explains the situation of the parent who perceived as segregation the school's attempts to provide additional but completely unnecessary support with the Finnish language for their Finnish-born child for the only reason of having an immigrant background. From the school's perspective, it was an attempt to provide additional support to a child from a perceived underprivileged background, while a participant deciphered it as segregation and a proof of inability to be fully integrated into Finnish society, even having been born there and speaking Finnish as a native language, as confirmed in a private conversation with the teachers. This was not a unique experience for the participant's children, which strengthened the participant's confidence about the inability to fully integrate being of an immigrant background. Another claim about inability to fully integrate concerned language bubbles formed by immigrants. This claim aligned with the findings of a European Commission survey (European Commission, 2016), which revealed that most teachers in Finland recognized the presence of foreign-language enclaves in schools. These enclaves hindered children from immigrant backgrounds from becoming fluent in Finnish and, consequently, from successfully integrating.

Regarding the connection between Finnish language skills and integration into Finnish society, both perspectives on successful integration discussed by the study participants have gained scientific attention—namely, that successful integration in Finland is attained either through mastering the Finnish language or, conversely, through adopting English as the main communication language. While the Finnish Board of Education emphasizes immigrants' proficiency in Finnish as crucial for social integration, enabling interaction within society, culture, and working life (OPH, 2022), other studies confirm that due to its unique role and prestige in Finland, including its well-established position as a medium of instruction at various educational levels, English can also serve as a means for integration for both adults and children (Leppänen and Nikula, 2007; Blommaert et al., 2012), with immigrants from high-income nations not being expected to learn Finnish as extensively as those lower in the hierarchy (Koskela, 2014). Nevertheless, skilled migrants often feel they cannot fully integrate into society, despite mastering the Finnish language. Instead of adopting Finnish customs, they are expected to continually represent their native cultures, showcasing cultural diversity within an international, multicultural community, which hinders their ability to truly integrate and feel a sense of belonging in Finnish society (Koskela, 2014).

## 6 Conclusion and limitations

This study investigated language choices and ideologies among seven transnational Hebrew-Russian participants relocating to Finland. The following ideologies were detected and formulated in this study: multilingual advantage, freedom of language choice, parental convenience, cultural and traditional value, and parental responsibility. The external ideology of language superiority prominent in their childhood was also discussed by the participants. The ideologies concerning code-switching and the connection between language knowledge and integration showed various patterns.

While advocating for multilingualism, participants maintained consistent language choices adopted in Israel after their relocation to Finland. The predominant concern was ensuring proficiency in at least one language, reflecting power dynamics and linguistic capital. Concerns over code-switching and the need for language purity at least in one language were evident, aligning with monolingual societal values. English emerged as crucial, with the education system largely responsible for its instruction. Views on the Finnish language as a means of social integration varied, with some emphasizing language mastery a key factor, while others questioned its sufficiency, reflecting broader societal debates on immigrant integration.

This study reflects how individuals and families interpret and manage their linguistic environments based on their beliefs, values, and experiences. Language ideologies shape and are shaped by the ways people perceive the roles and functions of different languages within their lives and communities. The study contributes to the theory of language ideology by addressing an new combination of languages—Hebrew, Russian, and Finnish—within transnational and multilingual contexts, offering a nuanced understanding of how language ideologies influence and are influenced by the process of relocation and integration. The unique combination of languages emphasizes the specific challenges and strategies employed by families managing multiple heritage languages and integrating into a new linguistic environment. Understanding this combination contributes to a more comprehensive view of language ideologies in diverse multilingual settings. The study findings also have implications for informing language policy and educational practices. By highlighting the language ideologies of transnational families, this research can guide policymakers and educators in developing strategies that better support multilingual families, address their specific needs, and foster effective language integration and maintenance practices. As described in Section 3.2., this study has limitations due to its small sample size and potential selection bias, which affect the generalizability of the findings. Supplementing the research with participant observations or longitudinal studies could provide additional insights and deepen the understanding of the findings. Lastly, to address research gaps effectively, future studies should strive for a more diverse sample of parents, as well as various linguistic environments, namely, different countries for relocation. This would ensure a broader representation of Generation 1.5 FSU-born Israeli parents' ideologies, especially in countries with larger Israeli communities.

## Data Availability

The data analyzed in this study is subject to the following licenses/restrictions: the data are not publicly available. Portions of the data that do not compromise participant anonymity are available upon request from the author. Requests to access these datasets should be directed to Gali Bloch, gali.bloch@helsinki.fi.

## References

[B1] Aitchison J. (1991). Language Change: Progress or Decay? 2nd Edn. Cambridge; New York, NY: Cambridge University Press (Cambridge approaches to linguistics).

[B2] Altman C. Burstein Feldman Z. Yitzhaki D. Armon Lotem S. Walters J. (2014). Family language policies, reported language use and proficiency in Russian – Hebrew bilingual children in Israel. J. Multiling. Multicult. Dev. 35, 216–234. 10.1080/01434632.2013.852561

[B3] Ballinger S. Brouillard M. Ahooja A. Kircher R. Polka L. Byers-Heinlein K. (2020). Intersections of official and family language policy in Quebec. J. Multiling. Multicult. Dev. 43, 614–628. 10.31234/osf.io/bjxm8 PMC11424030 39328886

[B4] Barron-Hauwaert S. (2004). Language Strategies for Bilingual Families: The One-Parent-One-Language Approach, 1st Edn. Bristol; Buffalo; Toronto, OM: Multilingual Matters.

[B5] Benor S. Krasner J. B. Avni S. (2020). Hebrew Infusion: Language and Community at American Jewish Summer Camps. New Brunswick, NJ: Rutgers University Press.

[B6] Bilaniuk L. (1997). “Speaking of Surzhyk”: ideologies and mixed languages. Harvard Ukrain. Stud. 21, 93–117.

[B7] Bloch G. (2024). Heritage Hebrew in Finland: Insights from Multilingual Families. Languages 9:216. 10.3390/languages9060216

[B8] Blommaert J. Leppänen S. Pahta P. Räisänen T. (eds.). (2012). Dangerous Multilingualism. London: Palgrave Macmillan UK.

[B9] Braun A. Cline T. (2014). Language Strategies for Trilingual Families: Parents' Perspectives. Bristol: Multilingual Matters (Parents' and teachers' guides 17).

[B10] Cangelosi M. Borghetti C. Bonifacci P. (2024). How parents' perceived value of the heritage language predicts their children's skills. Languages 9:80. 10.3390/languages9030080

[B11] Chiswick B. R. Gindelsky M. (2016). Determinants of bilingualism among children: an econometric analysis. Rev. Econ. Househ. 14, 489–506. 10.1007/s11150-015-9301-1

[B12] Cho E. K. Chen D. W. Shin S. (2010). Supporting transnational families. J. Natl. Assoc. Educ. Young Child. 65, 30–37. Available at: http://www.jstor.org/stable/42731076

[B13] Curdt-Christiansen X. L. (2009). Invisible and visible language planning: ideological factors in the family language policy of Chinese immigrant families in Quebec. Lang. Policy 8, 351–375. 10.1007/s10993-009-9146-7

[B14] De Houwer A. (2021). Bilingual Development in Childhood. 1st Edn. Cambridge: Cambridge University Press.

[B15] DellaPergola S. Lustick I. S. (2011). Israeli immigration/emigration. Isr. Stud. Rev. 26, 1–27. 10.3167/isr.2011.260202

[B16] Dockrell J. E. Papadopoulos T. C. Mifsud C. L. Bourke L. Vilageliu O. Bešić E.. (2022). Teaching and learning in a multilingual Europe: findings from a cross-European study. Eu. J. Psychol. Educ. 37, 293–320. 10.1007/s10212-020-00523-z

[B17] Dołowy-Rybińska N. Hornsby M. (2021). “Attitudes and ideologies in language revitalisation,” in Revitalizing Endangered Languages. 1st Edn, eds. J. Olko, and J. Sallabank (Cambridge: Cambridge University Press), 104–126.

[B18] Donitsa-Schmidt S. (1999). Language Maintenance or Shift: Determinants of Language Choice Among Soviet Immigrants in Israel (Doctoral dissertation). Toronto, ON: Ontario Institute for Studies in Education of the University of Toronto.

[B19] European Commission (2016). Finland: Concentrating Immigrants in the Same Areas crEates Problems in Schools. Available at: https://migrant-integration.ec.europa.eu/news/finland-concentrating-immigrants-same-areas-creates-problems-schools_en (accessed September 23, 2024).

[B20] Farella J. Moore J. Arias J. Elliott-Engel J. (2021). Framing indigenous identity inclusion in positive youth development: proclaimed ignorance, partial vacuum, and the peoplehood model. J. Youth Dev. 16, 1–25. 10.5195/jyd.2021.1059

[B21] Fogle L. W. King K. A. (2013). Child agency and language policy in transnational families. Iss. Appl. Linguist. 19:5288. 10.5070/L419000528838784612

[B22] Gal S. Irvine J. T. (2019). Signs of Difference: Language and Ideology in Social Life. 1st Edn. (Cambridge: Cambridge University Press).

[B23] Grosjean F. (1982). Life With Two Languages: An Introduction to Bilingualism. Cambridge, MA: Harvard University Press.

[B24] Haque S. (2011). Migrant family language practices and language policies in Finland. Apples J. Appl. Lang. Stud. 5, 49–64. 10.5281/zenodo.2008567

[B25] Hary B. Wein M. J. (2013). Religiolinguistics: on Jewish-, Christian- and Muslim-defined languages. Int. J. Soc. Lang. 2013, 85–108. 10.1515/ijsl-2013-0015

[B26] Helman A. (2002). “Even the Dogs in the Street Bark in Hebrew”: National Ideology and Everyday Culture in Tel-Aviv. Jewish Q. Rev. 92:359. 10.2307/1455449

[B27] Hirsch T. Lee J. S. (2018). Understanding the complexities of transnational family language policy. J. Multiling. Multicult. Dev. 39, 882–894. 10.1080/01434632.2018.1454454

[B28] Hoffman K. E. (ed.). (2007). We Share Walls: Language, Land, and Gender in Berber Morocco. 1st Edn. Hoboken, NJ: Wiley.

[B29] Idaryani I. Fidyati F. (2022). The impact of parental language ideology and family language policy on language shift and language maintenance: bilingual perspective. EduLite 7:192. 10.30659/e.7.1.192-208

[B30] King K. A. (2000). Language ideologies and heritage language education. Int. J. Biling. Educ. Biling. 3, 167–184. 10.1080/1367005000866770538754035

[B31] Kopeliovich S. (2010). Family language policy: a case study of a Russian-Hebrew bilingual family: toward a theoretical framework. Diaspora Indig. Minor. Educ. 4, 162–178. 10.1080/15595692.2010.490731

[B32] Korpela M. (2023). Under the Radar – Expatriate Children and Integration in Finland. Barn forskning om barn og barndom i Norden 41:5297. 10.23865/barn.v41.5297

[B33] Koskela K. (2014). Boundaries of belonging: highly skilled migrants and the migrant hierarchy in Finland. J. Finnish Stud. 17, 19–41. 10.5406/28315081.17.1.2.03

[B34] Kroskrity P. V. (2010). “Language ideologies,” in Handbook of Pragmatics, eds. J.-O. Östman, and J. Verschueren (Amsterdam: John Benjamins Publishing Company), 1–24.

[B35] Kroskrity P. V. (2016). Some recent trends in the linguistic anthropology of native North America. Annu. Rev. Anthropol. 45, 267–284. 10.1146/annurev-anthro-102313-03004127579784

[B36] Kwon J. Martínez-Álvarez P. (2022). A young linguistic and cultural mediator: a case of trilingual siblings' interaction. Int. Multiling. Res. J. 16, 47–64. 10.1080/19313152.2021.1930647

[B37] Lanza E. (2021). The family as a space: multilingual repertoires, language practices and lived experiences. J. Multiling. Multicult. Dev. 42, 763–771. 10.1080/01434632.2021.1979015

[B38] Lee J. S. Suarez D. (2009). “Chapter 5. A synthesis of the roles of heritage languages in the lives of children of immigrants: what educators need to know,” in The Education of Language Minority Immigrants in the United States. Multilingual Matters, eds. T. G. Wiley, J. S. Lee, and R. W. Rumberger (Bristol: Channel View Publications), 136–171.

[B39] Leppänen S. Nikula T. (2007). Diverse uses of English in Finnish society: discourse-pragmatic insights into media, educational and business contexts. Multilingua 26, 333–380. 10.1515/MULTI.2007.017

[B40] Leybenson M. Gutman M. Yemini M. (2023). Parental logic among Russian-speaking two-step migrants. J. Fam. Stud. 29, 1764–1780. 10.1080/13229400.2022.2082315

[B41] Lizardo O. (2017). Improving cultural analysis: considering personal culture in its declarative and nondeclarative modes. Am. Sociol. Rev. 82, 88–115. 10.1177/0003122416675175

[B42] McEwan-Fujita E. (2010). Ideology, affect, and socialization in language shift and revitalization: the experiences of adults learning Gaelic in the Western Isles of Scotland. Lang. Soc. 39, 27–64. 10.1017/S0047404509990649

[B43] Meir N. Avramenko M. Verkhovtceva T. (2021). Israeli Russian: case morphology in a bilingual context. Russian J. Linguist. 25, 886–907. 10.22363/2687-0088-2021-25-4-886-907

[B44] Ministry of Economic Affairs and Employment (2020). Successful Integration of Immigrants is the Sum of Many Parts. Available at: https://valtioneuvosto.fi/en/-//1410877/maahanmuuttajien-onnistunut-kotoutuminen-on-monen-tekijan-summa?__cf_chl_tk=hWUY.C50yFyITZgMXp9jtg_eoVaAQKPStuj5ZbUSHZI-1722759903-0.0.1.1-4393 (accessed September 23, 2024).

[B45] Naiditch L. (2008). Trends in the development of the Russian language abroad: Russian language in Israel. Russian Linguist. 32, 43–57. 10.1007/s11185-007-9019-7

[B46] Niznik M. (2011). Cultural practices and preferences of “Russian” youth in Israel. Isr. Aff. 17, 89–107. 10.1080/13537121.2011.522072

[B47] Official Statistics Finland (2023). Population by Origin and Language. Available at: https://stat.fi/tup/suoluk/suoluk_vaesto_en.html#Population%20by%20origin%20and%20language,%202019 (accessed September 23, 2024).

[B48] OPH (2022). Oman Äidinkielen Opetus. Available at: https://www.oph.fi/fi/koulutus-ja-tutkinnot/oman-aidinkielen-opetus (accessed September 23, 2024).

[B49] Otwinowska A. Marecka M. Casado A. Durlik J. Szewczyk J. Opacki M.. (2021). Does L2 proficiency impact L2-L1 transfer while reading L1 collocations? Evidence from behavioral and ERP data. Front. Psychol. 12:673761. 10.3389/fpsyg.2021.673761 34658998 PMC8513778

[B50] Palviainen Å. Bergroth M. (2018). Parental discourses of language ideology and linguistic identity in multilingual Finland. Int. J. Multiling. 15, 262–275. 10.1080/14790718.2018.1477108

[B51] Park M. Y. (2022). Language ideologies, heritage language use, and identity construction among 1.5-generation Korean immigrants in New Zealand. Int. J. Biling. Educ. Biling. 25, 2469–2481. 10.1080/13670050.2021.1913988

[B52] Perelmutter R. (2018a). “Israeli Russian in Israel,” in Languages in Jewish Communities, Past and Present, eds. B. Hary, and S. B. Benor (Boston, MA: De Gruyter Mouton), 520–543.

[B53] Perelmutter R. (2018b). Globalization, conflict discourse, and Jewish identity in an Israeli Russian-speaking online community. J. Pragmat. 134, 134–148. 10.1016/j.pragma.2018.03.019

[B54] Phoenix A. Faulstich Orellana M. (2022). Adult narratives of childhood language brokering: learning what it means to be bilingual. Child. Soc. 36, 386–399. 10.1111/chso.12462

[B55] Prashizky A. (2020). Homeland holidays as anchors of immigrant identity: New Year (Novy God) celebration among young Russian Israelis. Soc. Ident. 26, 16–30. 10.1080/13504630.2019.1667761

[B56] Quirk E. Hadeed N. Byers-Heinlein K. (2024). Trilingual families' language strategies: potential predictors and effect on trilingual exposure. Int. J. Multilingual. 1–18. 10.1080/14790718.2024.2302100 PMC12119091 40443954

[B57] Remennick L. (2003). The 1.5 Generation of Russian immigrants in Israel: between integration and sociocultural retention. Diaspora 12, 39–66. 10.1353/dsp.2011.004334409987

[B58] Remennick L. Prashizky A. (2019). Generation 1.5 of Russian Israelis: integrated but distinct. J. Mod. Jewish Stud. 18, 263–281. 10.1080/14725886.2018.1537212

[B59] Remennick L. Prashizky A. (2023). “Hosts, not guests, in Israel”: younger generations of Russian Israelis on the path to active citizenship. J. Mod. Jewish Stud. 22, 522–542. 10.1080/14725886.2023.2174417

[B60] Roberge M. M. (2002). California's Generation 1.5 immigrants: what experiences, characteristics, and needs do they bring to our English classes? CATESOL J. 14, 107–129. Available at: http://www.catesoljournal.org/wp-content/uploads/2014/07/CJ14_roberge.pdf

[B61] Rumbaut R. D. Rumbaut R. G. (1976). “Self and circumstance: journeys and visions of exile,” in The Dispossessed: An Anatomy of Exile, ed. P. I. Rose (Louise W and Edmund J. Kahn Institute), 331–343.

[B62] Schieffelin B. B. Woolard K. A. Kroskrity P. V. (1998). Language Ideologies: Practice and Theory. New York, NY: Oxford University Press.

[B63] Schiffman H. F. (1998). Linguistic Culture and Language Policy. 1. publ. in paperback. London: Routledge (The politics of language).

[B64] Schwartz M. Kozminsky E. Leikin M. (2009). Socio-linguistic factors in second language lexical knowledge: the case of second-generation children of Russian-Jewish immigrants in Israel. Lang. Cult. Curric. 22, 15–28. 10.1080/07908310802504119

[B65] Seo Y. (2021). Parental language ideologies and affecting factors in bilingual parenting in Korea. Eng. Teach. 76, 105–124. 10.15858/engtea.76.1.202103.105

[B66] Shankar S. (2008). Speaking like a model minority: “FOB” styles, gender, and racial meanings among Desi Teens in Silicon Valley. J. Linguist. Anthropol. 18, 268–289. 10.1111/j.1548-1395.2008.00022.x

[B67] Silverstein M. (1979). “Language structure and linguistic ideology,” in J11e Elements, eds. P. Clyne, W. Hanks, and C. Hofbauer (Chicago, IL: Chicago Linguistics Society), 193–248.

[B68] Soler J. Zabrodskaja A. (2017). New spaces of new speaker profiles: exploring language ideologies in transnational multilingual families. Lang. Soc. 46, 547–566. 10.1017/S0047404517000367

[B69] Spolsky B. (2004). Language Policy / Bernard Spolsky. Cambridge: Cambridge University Press.

[B70] Spolsky B. (2012). Family language policy – the critical domain. J. Multiling. Multicult. Dev. 33, 3–11. 10.1080/01434632.2011.638072

[B71] Spolsky B. (2018). “Sociolinguistics of Jewish language varieties,” in Languages in Jewish Communities, Past and Present, eds. B. Hary, and S. B. Benor (Berlin: De Gruyter Mouton), 583–601.

[B72] Ulitsa N. Yona L. Gogonsky A. Roer-Strier D. (2020). “Child risk and protection: perceptions of one-and-a-half generation immigrant parents from the Former Soviet Union and Israeli Social Workers,” in Context-Informed Perspectives of Child Risk and Protection in Israel, eds. D. Roer-Strier, and Y. Nadan [Cham: Springer International Publishing (Child Maltreatment)], 171–196.

[B73] Warditz V. Meir N. (2024). Ukrainian–Russian bilingualism in the war-affected migrant and refugee communities in Austria and Germany: a survey-based study on language attitudes. Front. Psychol. 15:1364112. 10.3389/fpsyg.2024.1364112 38845768 PMC11154112

[B74] Woolard K. A. (2020). “Language ideology,” in The International Encyclopedia of Linguistic Anthropology, 1st Edn., ed. J. Stanlaw (London: Wiley), 1–21.

[B75] Zacheus T. Kalalahti M. Varjo J. Saarinen M. Jahnukainen M. Mäkelä M. L.. (2019). Discrimination, harassment and racism in finnish lower secondary schools. Nord. J. Migrat. Res. 9:81. 10.2478/njmr-2019-0004

[B76] Zhang-Wu Q. (2024). Monolingual disobedience, multilingual guilt?: an autoethnographic exploration of heritage language maintenance during COVID-19 lockdowns. Multilingua 43, 267–287. 10.1515/multi-2023-0020

